# Endocytic Recycling of MHC Class I Molecules in Non-professional Antigen Presenting and Dendritic Cells

**DOI:** 10.3389/fimmu.2018.03098

**Published:** 2019-01-07

**Authors:** Sebastian Montealegre, Peter M. van Endert

**Affiliations:** ^1^Institut National de la Santé et de la Recherche Médicale, Unité 1151, Paris, France; ^2^Université Paris Descartes, Faculté de Médecine, Paris, France; ^3^Centre National de la Recherche Scientifique, UMR8253, Paris, France

**Keywords:** major histocompatibility, endosome, dendritic cell, recycling, antigen presentation, cross-presentation, Arf6, Rab11

## Abstract

Major histocompatibility complex class I (MHC I) molecules are glycoproteins that display peptide epitopes at the cell surface of nucleated cells for recognition by CD8^+^ T cells. Like other cell surface receptors, MHC class I molecules are continuously removed from the surface followed by intracellular degradation or recycling to the cell surface, in a process likely involving active quality control the mechanism of which remains unknown. The molecular players and pathways involved in internalization and recycling have previously been studied in model cell lines such as HeLa. However, dendritic cells (DCs), which rely on a specialized endocytic machinery that confers them the unique ability to “cross”-present antigens acquired by internalization, may use distinct MHC I recycling pathways and quality control mechanisms. By providing MHC I molecules cross-presenting antigens, these pathways may play an important role in one of the key functions of DCs, priming of T cell responses against pathogens and tumors. In this review, we will focus on endocytic recycling of MHC I molecules in various experimental conditions and cell types. We discuss the organization of the recycling pathway in model cell lines compared to DCs, highlighting the differences in the recycling rates and pathways of MHC I molecules between various cell types, and their putative functional consequences. Reviewing the literature, we find that conclusive evidence for significant recycling of MHC I molecules in primary DCs has yet to be demonstrated. We conclude that endocytic trafficking of MHC class I in DCs remains poorly understood and should be further studied because of its likely role in antigen cross-presentation.

## Introduction

MHC I molecules present pathogen, tumor and self-antigens to CD8+ T cells through the endogenous or direct and the exogenous or cross-presentation (1, 2) pathways. The spatio-temporal separation of these pathways (3) implies that intracellular transport of MHC-I molecules must be regulated, and that MHC I trafficking may vary according to cell type and particularly to the presence or absence of cross-presentation capacity. Mechanisms regulating trafficking likely are intertwined with mechanisms of quality control and act at various places in the cell: the endoplasmic reticulum (ER), the Golgi apparatus, the cell surface, and the endosomal system. A vast amount of information is available about the quality control steps selecting properly folded class I molecules in the secretory pathway (4–7). In contrast, the endocytic transport of class I molecules and the mechanisms of quality control in it are much less understood.

Analysis of endocytic trafficking is complicated by the fact that MHC I molecules exist in various forms presumably sensed by mechanisms of quality control, and that may follow distinct intracellular trafficking pathways: trimers made of a heavy chain, beta-2 microglobulin (β_2_m), and high affinity peptides; trimers made of heavy chain, β_2_m, and low affinity peptides; dimers without any bound peptides; and free heavy chains (FHC). For simplicity, we will refer to the trimers with high-affinity peptide as “fully conformed,” and the other forms as “sub-optimally loaded,” unless otherwise specified. Distinguishing complexes with high and low affinity peptides is important, since the affinity of the peptide-MHC interaction is the first determinant of the lifetime of class I molecules at the cell surface (8–10). It also determines the dissociation of β_2_m, the binding of which acts as signal preventing degradation of class I complexes (9). The picture becomes even more complex considering that different class I allotypes have different half-lives at the cell surface (11). Thus, putative quality control mechanisms should sense correctly structural variants for more than 5,500 class I allotypes (12) to discriminate between degradation and recycling.

As we will describe in detail, a large amount of information about the recycling pathways followed by class I molecules has been obtained in HeLa cells and H-2L^d^-expressing L cell fibroblasts. Available data suggest quantitative and mechanistic differences relative to the speed and efficacy of class I recycling between model cell lines. In contrast, very limited information is available on MHC I trafficking in professional antigen presenting cells (pAPCs). It is ironic that recycling has mainly been studied in cell lines unable to cross-present, given that the likely biological role of recycling concerns cross-presentation in DCs priming T cell responses to tumors and pathogens.

While some discrepancies between published studies may be derived from methodological approaches, many will be due to variation between cell types studied. In this review we will not only emphasize differences between model cell lines and pAPCs, but also examine the methods that have been used to obtain quantitative data on recycling efficacy and kinetics. Moreover, we will highlight knowledge on trafficking of fully conformed and sub-optimally loaded class I molecules obtained studying non-immune, non-phagocytic cell lines. Finally, we will relate these observations to existing and lacking knowledge on MHC I trafficking in pAPCs and its role in antigen cross-presentation. As we discuss key data on endocytic trafficking and evidence for differential sorting of distinct MHC I conformers, it needs to be kept in mind that the molecular players and chaperones mediating quality control in this context remain unknown. We anticipate that identification of such players will be required to fully understand endocytic trafficking and recycling of class I molecules, and conclusively answer the questions discussed below.

## Endocytosis in a Nutshell

Proteins that are destined to be recycled to the cell surface need, by definition, first to be internalized from the cell surface. Internalization of cell surface components is a constitutive event in all cell types and important in nutrient uptake, signal transduction, cell adhesion, and in renewal and recycling of plasma membrane components, among others (2, 13). Internalization of membrane proteins requires the formation of endocytic vesicles delivering cargo to the cell. The formation of such vesicles can be mediated by clathrin, a protein forming a lattice around the newly generated vesicle in the form of triskelions (14), or can be clathrin-independent. Clathrin-mediated endocytosis (CME) and clathrin-independent endocytosis (CIE) are commonly distinguished as the two main routes of endocytosis (15, 16). Proteins that are internalized via CME typically harbor the motif Y-X-X-Φ at their cytosolic tail, where Φ is any bulky hydrophobic amino acid and X is any amino acid. MHC I molecules do not possess such a motif, although a sequence in the cytoplasmic tail of HLA-A and B molecules has been postulated to represent a non-canonical motif for CME endocytosis (17). CIE pathways are named according to the morphology of the vesicle coat or cargo (e.g., caveolae, or lipid raft endocytosis), or to key intracellular proteins regulating trafficking (18). One of the latter CIE pathways is named for the small GTPase Arf6 and has been widely described as the mechanism of endocytosis of MHC I molecules in model cell lines. In this pathway, hydrolysis of Arf6-GTP is required twice, first after internalization of cargo (19) to change the phosphoinositide composition of the early endosomes and allow fusion with EEA-1 vesicles (13, 19), and a second time to promote recycling from tubular recycling endosomes (described below) (19, 20). Regardless of the mode of internalization, after 5 to 10 min, internalized proteins arrive to a shared station, the early sorting endosomes (21, 22). Typical markers of the sorting endosomes, with a luminal pH in the range of 6.3 to 6.8, are the GTPase Rab5 and its effector EEA-1 (23). From these vesicles, internalized cargo can be sorted to late endosomes and lysosomes for degradation or be re-directed to the cell surface using various routes of recycling. At least three recycling pathways have been described for model cell surface receptors and in model cell lines: direct recycling from the sorting endosomes to the cell surface (“fast recycling”), the route followed by the transferrin receptor; transport from sorting endosomes to the Trans Golgi Network (TGN) and then to the cell surface (“retrograde transport”) (24); and transport from the sorting endosomes to the endocytic recycling compartment (ERC) and then to the cell surface (“slow recycling”). Fast recycling is in the order of 5–10 min (25), whereas slow recycling is in the order of 30–60 min (2). For an extensive review of the mechanistic regulation of each pathway we refer the reader to two excellent recent reviews (22, 26). For MHC I molecules, a fourth pathway from late endosomes to the surface (discussed below) has also been suggested. We will focus mainly on the pathway involving the ERC, the main route taken by recycling MHC I molecules in non-professional APCs according to literature data.

Recycling endosomes are dynamic, tubulo-vesicular structures of nearly neutral pH in charge of sorting and re-exporting internalized membrane material (2, 26). The most widely used though by no means exclusive marker of recycling endosomes is the small GTPase Rab11. Rab11 localizes mostly to a perinuclear region that defines the ERC. Rab11 also localizes to the TGN (27) and to vesicular structures. Rab22 is another small GTPase involved in endosomal membrane trafficking (28) found both in the ERC and in tubular recycling endosomes (TRE) (29). Activation of Rab22 is required for the formation of TREs from the ERC, and inactivation is required for the final fusion of the tubules with the surface (29). Apart from Rab22, the biogenesis and maintenance of the TRE is in part regulated by the actin regulatory redox enzyme MICAL-L1(30), the family of Eps15 homology domain-containing 1-4 proteins (EHD-1 to 4) (31), and the small GTPases Arf6 (see above), Rab35, and Rab8a (32, 33). MICAL-L1 is a central protein required for the *de novo* generation of TREs (32) since it can bind directly phospholipids, and can form tubules *in vitro* and *in vivo* (34). MICAL-L1 serves as a hub for multiple proteins to regulate the formation of the TREs: it binds to EHD-1 via Rab8, as well as to Arf6 or Rab35 (33). Arf6 positively regulates recycling by aiding to localize Rab8 to the forming TREs, as well as by activating phospholipase D and PIP5 kinase, thereby providing the necessary lipids for the generation of recycling vesicles (35). Rab35, on the other hand, works as a negative regulator of TRE formation, by binding to MICAL-L1 and promoting GTP hydrolysis of Arf6 by the GTPase activating protein (GAP) ACAP2 (35, 36).

## Recycling of Fully Conformed MHC Class I Molecules: Methods and Evidence

**Figure 1:**
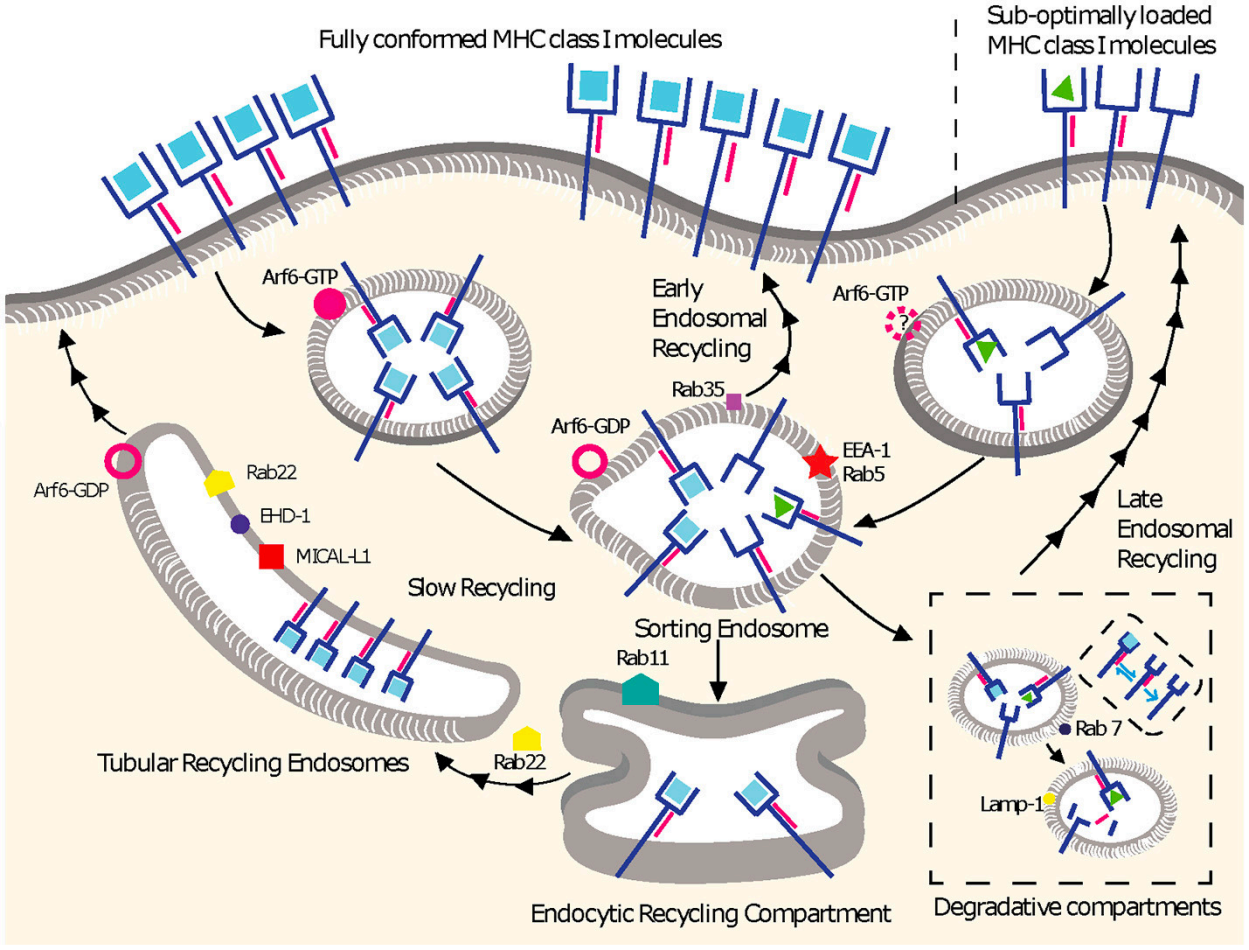
MHC I recycling pathways in non-professional APCs. Fully conformed and sub-optimally loaded class I molecules partition in different domains at the cell surface. Fully conformed class I molecules are internalized by CIE in vesicles decorated by Arf6-GTP and reach EEA1^+^/Rab5^+^ sorting endosomes. From here, they can follow an early endosomal recycling route regulated by Rab35 or enter into the slow recycling pathway. Upon arrival to the ERC, fully conformed molecules are incorporated into tubular recycling endosomes, which are formed by the proteins MICAL-L1, EHD-1, Rab8, Rab22, and Arf6. Vesicles derived from the tubular recycling endosomes finally fuse with the plasma membrane with the help of Arf6, delivering fully conformed class I molecules to the cell surface. Upon internalization, sub-optimally loaded class I molecules reach sorting EEA-1^+^/Rab5^+^ endosomes; the role of Arf6 in this is not known. From there, sub-optimally loaded class I molecules travel to degradative compartments, in which peptides and β_2_m will dissociate from the heavy chain, promoting the degradation of most molecules. However, a fraction may be able to recycle to the cell surface using a late endosomal recycling pathway independent of Rab11. Lines with single arrowheads represent internalization steps, whereas lines with multiple arrowheads represent recycling steps. Note that the ER is not represented.

Fully conformed and sub-optimally loaded MHC I molecules can be distinguished by monoclonal antibodies. We will first discuss work performed using antibodies recognizing the former category of class I molecules, which represents the vast majority of published studies (Figure 1). Early work from the groups of Watts and Jondal showed that upon internalization, class I molecules recycle to the cell surface (37, 38). Making use of a surface iodination assay in B lymphoblastoid cells, Reid and Watts were able to show that after accumulation of peptide-bound class I molecules in intracellular compartments by incubation with the inhibitor primaquine for 30 min, removal of primaquine resulted in recycling of nearly all the internalized class I to the cell surface within 16 min. Using the TAP-deficient thymoma cell line RMA-S, Abdel-Mottal et al. loaded class I molecules with glycopeptides and antibodies against the glycopeptide, then allowed for internalization, removed the remaining cell-surface complexes by a protease, and finally found that, depending on the peptide sequence, 36 to 63% of the class I molecules bound to the glycopeptides recycled to the surface. The re-appearance of the class I molecules was sensitive to chloroquine and leupeptin, indicating trafficking of the complexes via endosomal compartments. Although these experiments were performed using the thymoma line RMA-S, the findings of Abdel-Mottal et al. were a first indication that internalized peptides, and by extrapolation possibly antigens, might be loaded on recycling MHC I molecules in a “vacuolar” pathway.

Other work corroborated the conclusions of these early studies that class I molecules can recycle to the cell surface in model cell lines. The seminal work by Radakhrishna and Donaldson (20) showed for the first time the involvement of the small GTPase Arf6 in the recycling of MHC I molecules. As in most of the pertinent literature, the experimental system was HeLa cells, and the antibody used to detect class I was W6/32, which detects HLA heavy chains bound to β_2_m. In this system, class I localized to tubulo-vesicular structures decorated with Arf6. Moreover, in HeLa and Jurkat cells where the constitutively active mutant Arf6 Q67L was overexpressed, internalized class I molecules accumulated in PIP_2_ rich endosomes, thereby preventing their further degradation or recycling (13, 39, 40). Furthermore, overexpression of an effector domain mutant of Arf6 (N48I) in HeLa cells decreased recycling of class I molecules by 60% relative to control cells (35).

Subsequent studies of class I recycling in HeLa cells showed that 10–15 min after internalization, class I molecules reached tubular structures, presumably the TRE, the formation of which required EHD-1 among other players (see above) (29, 41). The quantitative assay to examine the role of EHD-1 in the recycling of class I was a “CELISA”: the authors seeded HeLa cells onto ELISA plates, incubated them with biotinylated MHC I antibodies at 37°C for 5 min, washed out free antibodies, and incubated the cells at 37°C for various time points. When EHD-1 was overexpressed, the number of cell surface class I molecules bound by biotinylated antibodies increased by 50% relative to the cell surface population present at the end of internalization (41).

The description of the role of Rab22 in endocytic recycling introduced the most widely used recycling assay so far (29). In a seminal study, HeLa cells were pulsed with mAb W6/32, allowed to internalize for 30 min at 37°C, acid-stripped to remove remaining cell surface complexes, and then chased at 37°C for various lengths of time. Quantification was based on the re-appearance of class I at the cell surface in unpermeabilized cells, as detected with a secondary antibody, compared to the internal class I signal, which was detected by removing the recycled class I with a second acid treatment, permeabilization of the cells, and detection with a secondary antibody. The read-out used microscopy or flow cytometry. In untransfected cells, 30% of the internalized class I population recycled to the surface by 30 min (29). Overexpression of wt Rab22 reduced recycling by 50%, an inhibitory effect that became even more pronounced upon overexpression of the dominant negative Rab22 S19N and the constitutively active Rab22 Q64L mutants. Using the same experimental system, the authors found that overexpression of the dominant negative mutant Rab11 S25N reduced class I recycling by nearly 80% relative to control cells, confirming a role for Rab11 in class I recycling in HeLa cells.

More recently, using the same recycling assay and HeLa cells, it was shown that the enzyme diacylglycerol kinase alpha (DGKα) was required for formation of tubular recycling endosomes by interacting with MICAL-L1 and generating phosphatidic acid (42). In turn, knockdown of DGKα delayed recycling of class I. Remarkably, after 30 min of internalization and 3 h of chase (recycling), the authors detected up to 40% recycled class I in wt Hela cells.

A putative alternative class I recycling pathway is mediated by Rab35, experimentally demonstrated in Cos-7 cells. Knockdown of Rab35 resulted in formation of enlarged EHD-1 negative endosomes (43). The authors proposed that Rab35 mediates “fast” direct recycling of class I from early endosomes to the cell surface, in a pathway distinct from the Rab22-Rab11 recycling axis. However, this conclusion was based on an assay that does not provide unequivocal evidence for recycling. The assay consisted in two 20-min incubations each followed by acid stripping to remove cell surface class I molecules. The first stripping removed MHC I not internalized after the initial 20-min pulse with an anti-MHC I antibody, and the second molecules “recycled” to surface after the second 20-min period. An increase in the number of labeled MHC-I molecules, detected by staining of permeabilized cells with a secondary antibody, was then interpreted as intracellular retention and lack of recycling. However, a role of Rab35 in degradation of internalized MHC-I molecules would equally well explain the increase by 60% of “retained” class I observed upon Rab35 knockdown.

Reviewing the different assays, some discrepancies are apparent, which may be biological or methodological. While the role of the different endocytic regulators is undisputed in the cell lines evaluated, the reported recycling kinetics of fully conformed class I molecules vary significantly from assay to assay and from cell line to cell line. The biochemical surface labeling assay (37) led to the conclusion that almost all fully conformed class I molecules that are internalized recycle very efficiently and fast. In contrast, the microscopy and FACS-based assays suggest that recycling of fully conformed class I recycling is slow and inefficient (Table 1). Perhaps the simplest explanation is that fully conformed class I can recycle via two different pathways, a fast one and a slow one. However, among the published pathways reviewed here, the “fastest” recycling pathway described implicates Rab35. In this pathway, recycling was detected after 20 min, which is already in the range of the slower recycling pathway dependent on the Arf6-Rab22-Rab11-MICAL-L1 axis. Thus, there is presently no conclusive evidence for class I recycling through a truly fast pathway returning for example the transferrin receptor to the surface. Variations between cell lines may also play a role. For example, in CHO cells, the Arf6 pathway plays a role in the recycling of the transferrin receptor, which is normally endocytosed via clathrin-mediated endocytosis (15), indicating that different recycling pathways might operate in different cells. Also, Rab22 is necessary for internalization of class I in Jurkat but not in HeLa cells (29, 40). As mentioned above, published data show remarkable variation with respect to the kinetics of class I recycling. Thus, in HeLa cells, the model system most frequently, recycling of W6/32 positive molecules ranges from 30 min to 3 h. At the same time, the authors of various publications agree on an estimated rate of 30–40% recycling class I molecules using the microscopy-based assay. How can the same rate of recycling be obtained in such divergent time spans? One possible explanation would be a mechanism of quality control that keeps the number of recycling class I molecules on the cell surface at any time below a certain threshold. For example, fully conformed class I molecules reaching the cell surface using the Arf6-Rab22-Rab11-MICAL-L1 axis within 30 min could rapidly be internalized again. It is unknown how many rounds of recycling a single class I molecule can undergo. It is also unclear whether and to what extent a peptide exchange occurs during class I recycling in non-professional APCs.

**Table 1:** Published data on MHC-I recycling.

**Allele**	**Cell type/line**	**Assay**	**Percent recycled of internalized pool**	**Time of maximal recycling**	**References**	**Observations**
HLA-A/B/C	EBV A46 B lymphoblastoid	Surface biotinylation	100	16 min	(37)	First demonstration that MHC I proteins to recycle upon internalization.
H-2D^b^	HeLa cells	CELISA	50	15 min	(41)	Recycling observed upon overexpression of EHD-1.
HLA-A/B/C	HeLa cells	mAb uptake- Microscopy	30	60 min	(29)	Rab22 overexpression decreases recycling to 50% relative to untransfected cells. Rab22 mutant overexpression reduces recycling to 15-25% relative to untransfected cells.
H-2L^d^, folded	L-L^d^ cells	mAb uptake-FACS	30	20 min	(44)	Recycling of unfolded H-2L^d^ was not detected with this assay. However, the authors could detect it in Mahmutefendić et al. (46) with a modification of the assay.
H-2L^d^, folded	L-L^d^ cells	mAb uptake-FACS	25–30	20 min	(45)	Recycling of several folded MHC I molecules was evaluated. The authors did not observe recycling of unfolded molecules.
H-2L^d^, folded	HeLa-L^d^	mAb uptake-FACS				
HLA-CW6	J26Cw6	mAb uptake-FACS				
HLA-B7	J26B7	mAb uptake-FACS			
HLA-A/B/C	HeLa cells	mAb uptake-microscopy	40	3 h	(42)	The absence of the enzyme diacylglycerol kinase alpha inhibits recycling of folded human class I molecules.
H-2L^d^, unfolded	L-L^d^ cells	mAb uptake-FACS	15–20	30 min	(46)	To observe recycling of unfolded H-L^d^ from late endosomes, the authors accumulated antibody-antigen complexes for 3h in late endosomal compartments, and then proceeded to evaluate recycling.
H-2K^b^, folded	JAWS DCs	mAb uptake-FACS	40	40 min	(47)	First quantitative demonstration of MHC I recycling in a DC-like cell line. Knocking down Rab22 reduces recycling efficiency to 10% of the internalized pool.

*The superscript letter indicates the allele of the gene indicated by the capital letter preceding it - no explanation required for any immunologist*.

Differences between class I allotypes might also lead to diversion into a different recycling pathway. In the secretory pathway, where the molecular players mediating quality control such as tapasin and calreticulin are well characterized, class I polymorphism is well known to affect quality control. For example, class I allotypes differing by a single amino acid, such as HLA-B^*^44:02 and B^*^44:05 or HLA-B^*^27:05 and B^*^27:09, differ greatly with respect to dependence on tapasin in order to acquire high affinity peptide ligands and leave the ER (48, 49). Similar differences might be revealed in endocytic quality control once the relevant chaperones will be identified. One candidate for such a role is the tapasin homolog TAPBPR, which can operate in a pH range of 6.0–7.2 and may therefore be able to mediate peptide exchange in endosomes. Interestingly, very recent data suggest that TAPBPR also acts in an MHC I allele-specific manner (50). In conclusion, the recycling pathways of fully conformed class I molecules in non-professional APCs still require clarification and additional investigation.

## Recycling of Sub-Optimally Loaded MHC Class I Molecules

Heavy chain-β_2_m empty dimers and FHC constitute a minor population out of the total pool of class I molecules present at the cell surface and can be identified by a number of monoclonal antibodies. The precise relative proportion of FHC and empt*y* dimers among surface class I molecules are not known but likely vary according to the cell type and state as well as to the class I allotype considered. For example, among the two murine allotypes studied most frequently, H-2K^b^ and H-2L^d^, cell surface L^d^ is known to comprise a larger proportion of FHC due to its lower stability relative to K^b^. FHC have been found at the cell surface of T and B lymphocytes under inflammatory conditions (51, 52) and in β_2_m deficient cells at resting conditions (53). While the function of the FHC is starting to be elucidated and may reside mainly in their binding to particular KIR receptors on NK cells (54–58), there is limited mechanistic evidence about their endosomal regulation and recycling.

Recent results provide initial insight into how sub-optimally loaded dimers are internalized, recycled, and degraded. The best evidence comes from the work of Lucin and co-workers, who have systematically evaluated the constitutive internalization of the murine class I allotype H-2L^d^ in their fully conformed and sub-optimally loaded forms (44, 59), as well as the early and late endosomal recycling of H-2L^d^ molecules (45, 46). This allotype is particularly suited for studying the different class I conformers because of the availability of the monoclonal antibodies 30.5.7 and 64.3.7, originally produced by Hansen et al. with a well-studied and exquisite specificity for the two conformers (60–62). As mentioned above, H-2L^d^ is less stable than other allotypes, resulting in a higher proportion of sub-optimally loaded or empty molecules, adding another argument in support of studying trafficking of different class I conformers using L^d^ as a model. The experimental system established by this group consists mainly, but not only, in L cell fibroblasts expressing L^d^, which constitutively express high levels of sub-optimally loaded dimers; monoclonal antibodies 30.5.7 and 64.3.7 distinguishing fully conformed class I trimers and β_2_m-bound heavy chains devoid of peptides, respectively; and a quantitative flow cytometry recycling assay based on the principle of the assay by Weigert et al. (29). Using these tools, they found that fully conformed internalized H-2L^d^ molecules that had accumulated during 1 h in a Rab11^+^compartment, presumably the ERC (45, 46), recycled with an efficiency of 20–30 %, reaching a plateau at 30 min. In contrast, sub-optimally loaded H-2L^d^ molecules were not detected in the Rab11^+^ ERC and could not recycle (44). They extended these results to other class I allotypes, such as HLA-Cw6 and HLA-B7, and cell lines, obtaining similar recycling rates (45). Whether recycling of fully conformed H-2L^d^ molecules was Arf6 dependent or not was not investigated. However, considering the recycling rates matching those observed in some of the HeLa-W6/32 recycling assays, and the involvement of the Rab11^+^ ERC, it is likely that they followed the Arf6 pathway. Since recycling of sub-optimally loaded class I molecules was not detected using the conventional methods, they modified the assay by internalizing sub-optimally loaded molecules bound to 64.3.7 for 3 h instead of 1 h, and comparing the signal for internalized vs. total labeled class I by flow cytometry (46). Surprisingly, they were able to detect recycling sub-optimally loaded molecules that had passed through Rab7^+^ late endosomes, with an efficiency of 15–20% after 30 min of chase.

So far, this is the sole quantitative evidence for recycling of sub-optimally loaded class I molecules, which may implicate a special pathway originating from late endosomes. In TAP-deficient fibroblasts pre-incubated at 26°C bearing relatively large numbers of sub-optimally loaded class I molecules at the cell surface (63, 64), FHCs, but not dimers, are rapidly removed from the cell surface (9). It is conceivable that a late endosomal pathway provides recycling dimers at low temperature, which at physiological temperature are degraded upon dissociation of β_2_m (9). Importantly, the cellular compartment where class I molecules are sorted for a round of recycling using the ERC-dependent recycling pathway or the late endosomal recycling pathway is not known. Whether these processes depend on Arf6, or whether they can be extrapolated to other cell lines or cell types, are other open questions.

## MHC Class I Recycling and Antigen Presentation

While MHC I recycling has been subjected to some cell-biological scrutiny, there is surprisingly little published evidence on its role in antigen presentation. Given that the endocytic pathway plays a mandatory role in cross-presentation of internalized antigens, it may not surprise that the available literature concerns exclusively this pathway. Indeed, a role of recycling class I molecules in peptide loading of class I molecules in the ER appears little likely. However, class I molecules can also be loaded with endogenous peptides in post-ER compartments potentially accessible for recycling class I molecules. For example, Hsc70-coupled endogenous antigens can be processed through chaperone mediated autophagy (65), and HSV-1 antigens through a non-canonical pathway of macro-autophagy (66), both presumably implicating antigen degradation in endolysosomal compartments. Moreover, peptide fragments of endogenous transmembrane proteins can be produced and loaded in the same type of compartment (67). However, whether recycling MHC I molecules were responsible for presentation in these studies is not known.

**Figure 2:**
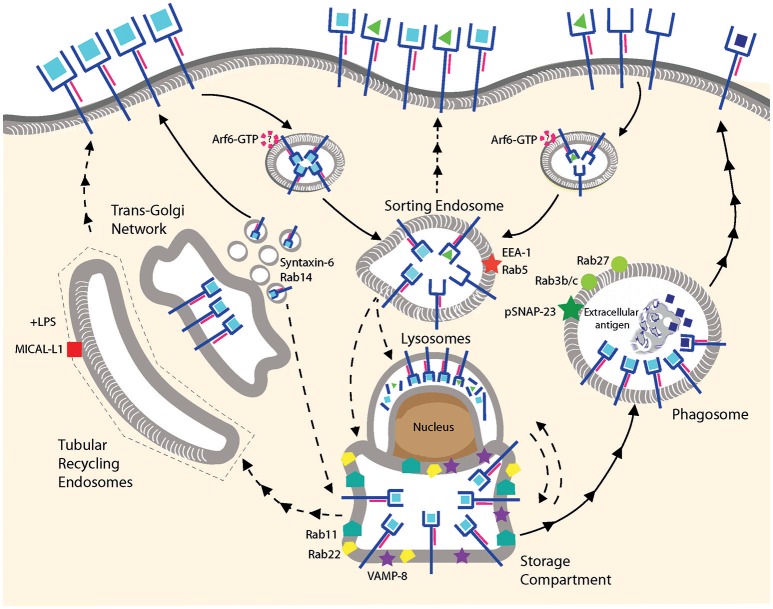
MHC I recycling in pAPCs. Fully conformed and sub-optimally loaded class I molecules are internalized and reach sorting endosomes presumably by CIE. It is not known whether Arf6 is involved for internalization of one or both conformers. Under resting conditions, fully conformed class I molecules reside in a post-ER compartment positive for Rab11/Rab22/VAMP8. Class I molecules might be provided to this compartment from the sorting endosomes, from the secretory pathway using TGN-derived vesicles positive for Syntaxin-6/Rab14, or from lysosomes. Given that the origin of the MHC I molecules in this compartment remains to be clarified, we prefer the term “storage compartment” to “ERC.” Upon stimulation by TLR ligands, fully conformed class I molecules are recruited from the storage compartment to phagosomes positive for Rab3b/c, Rab27, and pSNAP23, and then travel to the cell surface via an uncharacterized mechanism. Peptide exchange may occur in the phagosome although formal proof is lacking. Tubular recycling endosomes bearing MICAL-L1 have been observed upon stimulation with LPS, but class I molecules have not been detected so far within the tubules. Lines with single arrowheads represent internalization and lines with multiple arrowheads recycling steps. Dashed lines represent hypothetical transport steps. Note that the ER is not represented.

As discussed above, there appears to exist significant variation regarding the pathways and type of class I molecules able to recycle in non-immune cells. The scenario in pAPCs, e.g., cells derived through differentiation from monocytes or from bone marrow precursors *in vitro*, or primary DCs obtained from mice or humans is even less understood. In DCs but probably also some macrophage types capable of cross-presentation, class I molecules need to have access to peptides from internalized antigens. This can occur in the perinuclear ER through a cytosolic pathway of cross-presentation, or in non-ER compartments, following either the ER-phagosome pathway or vacuolar pathways (2). In the two latter scenarios, cross-presenting pAPCs may require an alternative source of class I molecules independent of the secretory pathway. It is in this context that recycling class I molecules emerge as a candidate source of MHC I molecules provided to the ER-phagosome and the vacuolar pathway of cross-presentation (Figure 2).

One of the first indications of potential pathways operating in professional antigen presenting cells [for the subtypes of DCs, the reader is referred to other reviews (68, 69)], was curiously obtained in a melanoma cell line. Grommé et al. fractionated MelJuSo melanoma cells and identified HLA class I molecules in a fraction also containing acidic HLA class II loading compartments. A significant proportion of HLA class I-peptide complexes remained stable and could be immunoprecipitated at pH = 5 using W6/32. This implied the existence of a compartment potentially acidic enough to promote peptide exchange, but not enough to degrade class I molecules (70). More direct evidence for peptide exchange in presumably recycling MHC I molecules was provided by studies on TAP-deficient macrophages. Pre-incubation with peptide ligands stabilized a pool of class I molecules on these cells which then allowed for cross-presentation of a bacterial antigen through a vacuolar pathway, most likely involving peptide exchange in an acidic compartment (71). Coming back to a more physiologic setting, various groups have reported the existence of a post-ER compartment containing presumably fully conformed class I molecules in different types of (TAP-sufficient) DCs (72–74). A common characteristic of the latter studies is that upon incubation with primaquine, cross-presentation of soluble antigens is blocked (3, 75, 76). A possible interpretation is that class I molecules are located in a mildly acid endosomal compartment from which they are unable to recycle upon inhibition of acidification by primaquine.

Do class I molecules recycle in pAPCs? And if so, do they use the pathways described in non-immune cell lines? The recent studies from Nair-Gupta et al. and Cebrian et al. built on the knowledge gained from the studies of non-immune cell lines to identify players in the cross-presentation pathways. The former study characterized a post-ER Rab11^+^/VAMP8^+^ compartment containing MHC I molecules in bone marrow-derived DCs (BM-DCs) and suggested that this compartment constituted an important source of cross-presenting MHC I molecules. Upon stimulation by TLR-2/4 ligands, class I molecules derived from the Rab11 compartment were recruited to phagosomes. Knockdown of Rab11 in BM-DCs had a profound detrimental effect on antigen cross-presentation. Remarkably, the authors showed that primary type 1 conventional DCs (cDC1), but not cDC2 harbor the class I/Rab11^+^ compartment, suggesting that cDC2s may have a different source of class I molecules for cross-presentation (77). It is puzzling that pDCs, which also have the post-ER compartment (73) have shown to be less efficient than their cDC1 and cDC2 counterparts in antigen cross-presentation.

Consistent with observations made in HeLa cells, the study by Cebrian et al. (47) showed that Rab22 partially co-localizes with Rab11 and with fully conformed class I molecules in BM-DCs and in JAWS-II cells, an immortalized cell line derived from C57BL/6 BM-DCs lacking p53. A 50% knockdown of Rab22 was sufficient to abolish the post-ER compartment containing class I molecules, as well as to compromise antigen cross-presentation. Rab22 knockdown not only hampered cross-presentation of soluble OVA but also of OVA secreted by Toxoplasma gondii into the parasitophorous vacuole of parasite-infected DCs. This result corroborated the importance of a Rab11+ compartment containing MHC I molecules in cross-presentation, however it remained unclear whether the MHC I molecules in this compartment actually derived from the cell surface. To study recycling, Cebrian and associates used an assay similar to that used by Allaire to study the role of Rab35 (43), in which an increase in MHC I molecules “retained” intracellularly was interpreted as evidence of lack recycling. As noted above, an intracellular accumulation of internalized MHC I molecules can be due both to compromised recycling and to reduced degradation. Thus, the finding that Rab22 knockdown inhibited “disappearance” of internalized MHC I over a 40-min period almost completely may indicate a role in MHC I recycling and/or in routing to a degradative compartment. Thus, in our view, MHC I recycling in murine DCs remains to be demonstrated conclusively.

Our review of the literature, as well as our own unpublished observations, suggests that monitoring MHC I recycling in primary DC-like populations, be they differentiated *in vitro* from bone marrow or monocytes, or isolated as primary *in vivo* DC populations, remains challenging. Considering the specialization but also plasticity of DCs, as well as the variation already existing between non-immune cell lines, it would not be surprising to find distinct recycling rates and pathways in primary DC populations. The role of recycling MHC I molecules might also be limited to specific antigen types. As an example, in a study including an assay directly measuring internalized MHC I molecules re-appearing at the cell surface, van den Eynde et al. recently showed that cross-presentation by human monocyte-derived DCs (mo-DCs) of a synthetic long peptide (an antigen type of interest for tumor vaccination) involved peptide exchange on MHC I molecules, however these molecules were nascent rather than recycling (78).

## Outlook

Although the cited papers provide solid evidence for an important role of Rab11 and Rab22 in cross-presentation, important mechanistic issues remain unresolved. Identification of the Rab11/Rab22/VAMP8 compartment containing fully conformed class I molecules may suggest that the class I molecules detected originate from the cell surface, where they return to through recycling. However, as discussed above, there is no formal proof that this is the case. Several alternative scenarios could be considered. The TGN might feed this compartment with fully conformed class I molecules arriving through the secretory pathway, using, for example, Syntaxin 6^+^ and Rab14^+^ TGN-derived vesicles (79, 80). Another hypothesis is the adaptation of the late endosomal recycling pathway described by Lucin and coworkers, where lysosomes would communicate with recycling endosomes that are in physical proximity, thereby providing an environment where class I molecules are sorted for peptide exchange and routing into the recycling pathway, or degradation.

Another issue that has not been well studied in pAPCs is the formation of tubular recycling endosomes. In the work by Cebrian et al. (47), Di Puchio et al. (73), Zou et al. (74), Nair-Gupta et al. (77), Croce et al. (81), such tubules were not described. The endocytic system of DCs remodels upon stimulation by TLR ligands, such that resting DCs do not behave like an LPS stimulated DC (82, 83). In this context, it appears that the formation of elongated tubules originating from the ERC in moDCs requires TLR stimulation and the formation of the immunological synapse (84, 85). The formation of the tubules requires the presence of MICAL-L1 (86), as in HeLa cells. However, as opposed to HeLa cells, class I molecules have not been observed in such tubules in pAPCs. The observations reported so far have made use of specimens treated with fixation, which renders visualization of such tubules difficult (26). Live cell-imaging methods might reveal the presence of such tubules in resting dendritic cells. Whether Arf6, Rab8, Rab35, ACAP1, or other proteins are required for the formation of such tubules remains to be investigated.

It is important to highlight that BM-DCs and moDCs, which have served as a model to study cross-presentation (but not class I recycling so far) *in vitro*, might not reflect the pathways of class I recycling and cross-presentation operating *in vivo* (87, 88). Unfortunately, the typical cell biological and cross-presentation assays require a substantial number of cells that can be difficult to obtain for primary pAPC subsets. Since BM-DCs and moDCs reflect most closely the properties of inflammatory dendritic cells and macrophages found *in vivo* (89), and the latter ones use in some cases a purely vacuolar cross-presentation pathway (90), it is important to study recycling of class I molecules in primary cells. We anticipate that there will be significant variation and plasticity in the recycling pathways and rates of class I molecules in primary pAPC populations. Identifying the molecular machinery in charge of endocytic quality control will be essential in order to fully decipher MHC I endocytic trafficking, recycling and cross-presentation. Finally, the functional impact of MHC I recycling is almost entirely unexplored. Previous studies have been limited to examining presentation of the model antigen OVA to a CD8+ T cell line. The ultimate challenge for the field will be explore the impact of MHC I recycling in T cell responses to pathogens and tumors.

## Author Contributions

All authors listed have made a substantial, direct and intellectual contribution to the work, and approved it for publication.

### Conflict of Interest Statement

The authors declare that the research was conducted in the absence of any commercial or financial relationships that could be construed as a potential conflict of interest.
